# Transcriptional Control of Vascular Smooth Muscle Cell Proliferation by Peroxisome Proliferator-Activated Receptor-γ: Therapeutic Implications for Cardiovascular Diseases

**DOI:** 10.1155/2008/429123

**Published:** 2007-12-06

**Authors:** Florence Gizard, Dennis Bruemmer

**Affiliations:** Division of Endocrinology and Molecular Medicine, University of Kentucky College of Medicine, Lexington, KY 40536, USA

## Abstract

Proliferation of vascular smooth muscle cells (SMCs) is a critical process for the development of atherosclerosis and complications of procedures used to treat atherosclerotic diseases, including postangioplasty restenosis, vein graft failure, and transplant vasculopathy. Peroxisome proliferator-activated receptor (PPAR) γ is a member of the nuclear hormone receptor superfamily and the molecular target for the thiazolidinediones (TZD), used clinically to treat insulin resistance in patients with type 2 diabetes. In addition to their efficacy to improve insulin sensitivity, TZD exert a broad spectrum of pleiotropic beneficial effects on vascular gene expression programs. In SMCs, PPARγ is prominently upregulated during neointima formation and suppresses the proliferative response to injury of the arterial wall. Among the molecular target genes regulated by PPARγ in SMCs are genes encoding proteins involved in the regulation of cell-cycle progression, cellular senescence, and apoptosis. This inhibition of SMC proliferation is likely to contribute to the prevention of atherosclerosis and postangioplasty restenosis observed in animal models and proof-of-concept clinical studies. This review will summarize the transcriptional target genes regulated by PPARγ in SMCs and outline the therapeutic implications of PPARγ activation for the treatment and prevention of atherosclerosis and its complications.

## 1. INTRODUCTION

Most cardiovascular diseases result from complications of atherosclerosis, which is a multifactorial process characterized by chronic inflammation, lipid accumulation, and the formation of a complex atherosclerotic lesion [1]. Recruitment of monocytes, their differentiation into macrophages, and uptake of LDL-derived cholesterol are the major cellular events contributing to early fatty streak formation [2, 3]. Continued intracellular cholesterol accumulation results in the generation of endogenous inducers of inflammatory and proliferative gene expression and a broad range of cellular and humoral responses contributing to lesion initiation and progression [4]. The resulting chronic inflammatory state and the enrichment of lipid-laden macrophages ultimately lead to the formation of a complex atherosclerotic lesion [5].

During the course of atherosclerotic lesion formation, secreted growth factors and cytokines promote the migration and proliferation of vascular smooth muscle cells (SMCs) to contribute to neointima formation [6]. This chronic proliferative response of SMCs promotes further lesion development through the production of proinflammatory mediators and the synthesis of extracellular matrix molecules, which is required for the retention of lipoproteins and often constitutes the majority of the protein content of the advanced lesion responsible for luminal obstruction [1]. However, SMC proliferation within the developing lesion may also exert beneficial effects by forming a fibrous cap covering the advanced atherosclerotic lesion, an important mechanism for the stability of the plaque [7]. The result of this chronic process is the development of an advanced atherosclerotic lesion, which may ultimately cause luminal obstruction and ischemic complications.

Once occlusive atherosclerotic disease has developed, the standard of care may include angioplasty, coronary artery bypass grafting, or cardiac transplantation. However, all current treatment approaches are limited by a varying degree of treatment failure and reocclusion of the arterial lumen. Among the cellular mechanisms responsible for this failure of the current interventional procedures used to treat occlusive atherosclerotic diseases, such as postangioplasty restenosis, transplant vasculopathy, and coronary artery bypass graft failure, SMC proliferation constitutes a prime mechanism [6]. In the past decade, elegant progress in interventional cardiology has provided the introduction of drug-eluting stents delivering rapamycin or paclitaxel into the vessel wall that target SMC proliferation [8]. However, despite initial enthusiasm, the complete inhibition of the healing response using these approaches may leave a thrombogenic vessel surface at risk of in-stent thrombosis and vessel occlusion [9]. Thus, despite these advances, ideal therapy for occlusive vascular disease is still far from established.

In an era marked by the increasing prevalence of obesity, diabetes, and cardiovascular disease, members of the nuclear hormone receptor superfamily have emerged as transcription factors that regulate diverse aspects of metabolism [10, 11]. In addition to their function to act as molecular sensors of lipid and carbohydrate homeostasis, several members of the nuclear hormone receptor family, including the peroxisome proliferator activated receptor (PPAR) γ, also exert beneficial pleiotropic effects to reduce atherosclerosis and its complications [12, 13]. PPARγ is the molecular target for the synthetic thiazolidinediones (TZD), such as rosiglitazone and pioglitazone, clinically used as insulin sensitizers in patients with type 2 diabetes [14]. Over the last decade, a wealth of evidence has supported a beneficial role for TZD PPARγ agonists in the regulation of vascular gene expression programs [12, 13]. While PPARγ expression itself is increased in response to vascular injury [15–17], its activation by TZD suppresses SMC proliferation through several mechanisms involving the regulation of genes encoding proteins in SMC migration [15], proliferation [15], differentiation [18], senescence [19], and apoptosis [16]. In the following review, we will discuss the role of PPARγ in vascular biology with respect to the control of proliferative gene expression programs that underlie SMC proliferation and the development of cardiovascular diseases.

## 2. PPARγ: A LIGAND-ACTIVATED TRANSCRIPTON FACTOR EXPRESSED IN VASCULAR CELLS

The detailed structure and molecular biology of PPARγ have previously been outlined in excellent review articles [11, 20]. Briefly, the PPAR subfamily of nuclear receptors consists of 3 isoforms, that is, PPARα (NR1C1), PPARβ (also known as δ, NR1C2), and PPARγ (NR1C3). PPARs regulate gene expression upon heterodimerization with the retinoid X receptor (RXR, or NR2B) and subsequent binding to specific response elements located in the promoter regions of target genes. Although presently there are no proven pathways for endogenous ligands in vivo, all PPARs are activated in vitro by fatty acids (FAs). PPARγ is activated by the prostaglandin D2 derivative 15-deoxy-Δ ^12, 14^-prostaglandin J2 (15d-PGJ2) [21] and forms of oxidized linoleic acid, 9- and 13(S)-HODE [22]. Synthetic PPARγ ligands include TZD, such as troglitazone, rosiglitazone, and pioglitazone, as well as non-TZD derivates. PPARγ is predominantly expressed in adipose tissue and has been characterized as an important regulator of adipocyte differentiation and glucose homeostasis [14]. Based on their efficacy to improve insulin sensitivity, the TZD PPARγ ligands rosiglitazone and pioglitazone are currently being utilized in clinical practice to treat insulin resistance in patients with type 2 diabetes [23, 24].

In addition to the metabolic effect of PPARγ, the receptor is expressed in atherosclerotic lesions [15, 25] and in all vascular cell types including endothelial cells (EC) [26], macrophages [27], T lymphocytes [28], and SMCs [29]. In EC, PPARγ is activated in response to atheroprotective laminar flow [30]. Ligand-induced activation of PPARγ in these cells suppresses the expression of genes responsible for the adhesion of monocytes to the endothelium (i.e., VCAM-1 [31, 32], ICAM-1 [33]) and their transendothelial migration [34], which are both crucial early processes for the subsequent development of atherosclerosis. In macrophage biology, PPARγ has been demonstrated to suppress inflammatory gene expression and to decrease intracellular lipid accumulation and foam-cell formation [35, 36]. Finally, increased PPARγ expression has been demonstrated in neointimal layers during atherosclerotic lesion development [15, 25]. Concomitant with the phenotypic shift from quiescent SMCs resident in the uninjured vessel wall to proliferating SMCs in the neointima, PPARγ expression is induced in the neointima following vascular injury [15, 16]. Considering the importance of SMC proliferation during atherosclerosis and its complications [6], this increased expression of PPARγ in neointimal SMCs has provided an important rationale to further exploit the role of PPARγ for the proliferative response that underlies the development of neointima formation and atherosclerotic cardiovascular diseases.

## 3. TRANSCRIPTIONAL REGULATION OF SMOOTH MUSCLE CELL PROLIFERATION BY PPARγ LIGANDS

**Figure 1:**
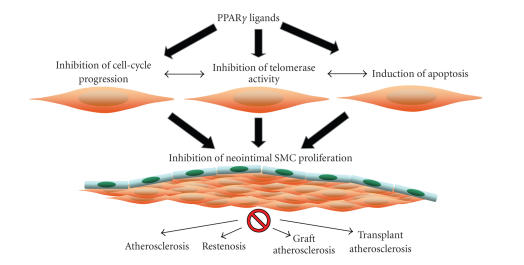
Cellular mechanisms involved in the inhibition of SMC proliferation by PPARγ during cardiovascular diseases. PPARγ regulates genes encoding proteins involved in diverse SMC processes including cell proliferation, cell-cycle progression, senescence, and apoptosis.

The physiological state of the SMCs in the arterial wall is determined by endogenous and exogenous signals, and often the endpoint that interpretates these signals is gene transcription [37]. Emerging evidence has implicated PPARγ as a key transcriptional modulator of SMC function. In the following section, we outline the role of PPARγ in the regulation of diverse SMC processes including cell proliferation, cell-cycle progression, senescence, and apoptosis (see Figure 1).

### 3.1. Regulation of SMC proliferation and cell-cycle progression by PPARγ agonists

**Figure 2:**
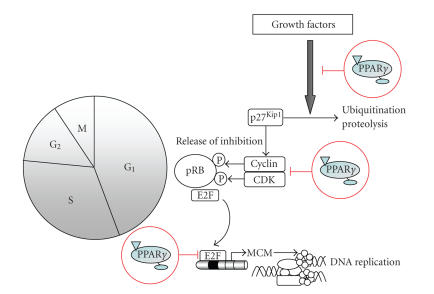
PPARγ targets cell-cycle progression. Phosphorylation of the retinoblastoma gene product (pRB) by specific G_1_ CDKs represents the critical checkpoint of the G_1_/S transition of the cell cycle. pRB phosphorylation releases E2F allowing the expression of genes required for DNA synthesis. By preventing the degradation of the CDK inhibitor (CDKI) p$27^{\text{Kip1}}$, PPARγ ligands inhibit mitogen-induced pRB phosphorylation and downstream expression of key E2F-regulated genes (i.e., MCM genes) responsible for the initiation of DNA replication.

Mitogenic growth factors secreted during vascular injury converge into a final common signaling pathway regulating the proliferative response of SMCs: the cell-cycle [6] (see Figure 2). While SMCs are in a quiescent state (G_0_) in the uninjured artery, they transit in response to mitogenic stimulation through the G_1_ phase of the cell-cycle and ultimately enter S phase to undergo replication [38]. Cell-cycle progression is under the control of cyclins and cyclin-dependent kinases (CDKs), which phosphorylate the retinoblastoma gene product (pRB) [39]. pRB phosphorylation represents the critical checkpoint of the ${\text{G}}_{1}\to \text{S}$ phase transition and increased pRB phosphorylation correlates with the induction of SMC proliferation in injured vessels [40, 41]. Consistent with this, maintenance of high levels of phosphorylated pRB is required for the development of intimal hyperplasia. Upon pRB phosphorylation, sequestered E2F transcription factors are released to induce the transcription of genes involved in the regulation of S phase DNA synthesis [42]. Through CDK-inhibitors (CDKI), including p$27^{\text{Kip1}}$, the activity of cyclin/CDK complexes in quiescent SMCs is inhibited providing a second layer of regulation [43, 44]. In response to mitogens, p$27^{\text{Kip1}}$ undergoes ubiquitination and degradation through the proteasome pathway allowing CDK/cyclin complexes to phosphorylate pRB [45]. Therefore, mitogen-induced degradation of p$27^{\text{Kip1}}$ is an initial requirement for pRB phosphorylation and subsequent ${\text{G}}_{1}\to \text{S}$ cell-cycle progression [46].

PPARγ ligands have been demonstrated in various studies to prevent mitogen-induced SMC proliferation and the mechanisms by which this inhibition of proliferation occurs appear to involve an arrest in the ${\text{G}}_{1}$ phase of the cell cycle [47–49]. The growth-inhibitory effects of PPARγ agonists were first associated with their ability to prevent mitogen-induced degradation of the CDKI cyclin-dependent kinase inhibitor (CDKI) p$27^{\text{Kip1}}$, which inhibits the activity of cyclin/CDK and consequently reduces the cellular levels of phosphorylated pRB [47]. Since in vivo gene transfer of p$27^{\text{Kip1}}$ significantly inhibits neointimal cell proliferation [43], p$27^{\text{Kip1}}$ likely constitutes an important target for the anti-proliferative effects of PPARγ activation. Consistent with its function to suppress the activity of cyclin/CDK-complexes, stabilization of p$27^{\text{Kip1}}$ by PPARγ ligands has been demonstrated to inhibit cyclin/CDK activity, an effect that ultimately translates into a prevention of mitogen-induced pRB phosphorylation [47].

DNA microarray analysis further identified that minichromosome maintenance proteins (MCM) 6 and 7 are inhibited by PPARγ ligands in SMCs [50]. MCM proteins represent bona fide E2F target genes [51] and play a central role in the regulation of the initiation of DNA replication ensuring that DNA replicates only once during cell cycle (for review see [52]). In eukaryotes, MCM2–MCM7 are recruited onto replication origins during the ${\text{G}}_{1}$ phase of the cell cycle and assembled into a heteromeric hexamer. Formation of this prereplication complex, a process often referred to as “replication licensing”, establishes the competence of this origin for the initiation of DNA replication in the subsequent S phase. Therefore, the inhibition of MCM gene expression by PPARγ ligands provides evidence that the inhibitory effects of PPARγ ligands on ${\text{G}}_{1}\to \text{S}$ transition are the result of targeting the pRB/E2F/MCM pathway.

### 3.2. PPARγ activation and induction of apoptosis in SMCs

In addition to the role of TZD in the regulation of ${\text{G}}_{1}\to \text{S}$ cell-cycle progression, several studies have demonstrated that TZD induce apoptosis in SMCs [16, 53, 54]. Among the regulated target genes mediating PPARγ-induced apoptosis is the growth-arrest and DNA damage-inducible gene 45 (GADD45) [53]. Molecular analyses demonstrated that PPARγ-induced GADD45 gene transcription is mediated through an Oct-1-dependent mechanism [53]. Although the exact function of GADD45 remains unclear, GADD45 has been implicated in growth suppression [55] and apoptosis [56, 57]. Through its association with Cdc2, GADD45 disrupts the interactions of Cdc2 with cyclin B1 and, thus, may induce G_2_/M arrest [58]. The GADD45 gene, therefore, may represent a unique target for drugs that induce cell-cycle arrest, apoptosis, and differentiation such as PPARγ ligands.

The second pathway that has been demonstrated to induce apoptosis by PPARγ ligands involves the induction of transforming growth factor (TGF)-β by PPARγ [54]. TGF-β is an essential cytokine involved in the control of the balance between proliferation and apoptosis in SMCs [59]. Previously, TZD-induced apoptosis of SMCs has been suggested to depend on the induction of TGF-β and subsequent downstream nuclear recruitment of phospho-Smad2 [54]. Interestingly, TGF-β-induced apoptosis is partly mediated by Smad-dependent expression of GADD45 [60]. Therefore, it is possible if not likely that GADD45 constitutes a key downstream mediator of apoptosis induced by PPARγ activation.

A third mechanism that has been implicated in PPARγ-induced SMC apoptosis involves the transcriptional induction of the interferon regulatory factor-1 (IRF-1), a transcriptional factor with anti-proliferative and proapoptotic properties. Lin et al. recently demonstrated that both TZD and PPARγ overexpression upregulate IRF-1 expression in SMCs [61]. Reducing IRF-1 expression by antisense approaches attenuated PPARγ-induced SMC apoptosis suggesting that the PPARγ-regulated IRF-1 pathway contributes to the proapoptotic effects observed with TZD.

### 3.3. Regulation of SMC telomerase and senescence by PPARγ ligands

Telomerase has been linked to multiple developmental processes including cell proliferation, senescence, and aging [62–64]. Telomeres, the DNA-protein complexes at the ends of chromosomes, are stabilized by the ribonucleoprotein telomerase reverse transcriptase (Tyyy3) to serve as protective capping and to prevent cellular senescence [65, 66]. In most adult cells Tyyy3 expression and telomerase activity are repressed and telomeres shorten during tissue renewal [67], and it has been proposed that this telomere exhaustion is rate limiting for lifespan [68]. Loss of telomere length beyond a critical threshold results in cellular senescence [59], a state in which cells are unresponsive to mitogenic stimuli [69]. These molecular features of telomerase to prevent senescence are highly conserved among eukaryotes and act on somatic cells as biological clock to ultimately result in permanent growth arrest and entry into replicative senescence [70].

In SMCs, telomerase activity is required for cell proliferation, and disruption of telomerase activity reduces atherosclerosis and neointima formation [71–73]. TERT is the limiting factor for telomerase activation in response to mitogenic stimuli and TERT antisense oligonucleotides inhibit SMC proliferation [71, 72]. This suggests that TERT may play an important role in the regulation of SMC proliferation and neointima formation. A recent study demonstrated that mitogen-induced telomerase activity in SMCs is inhibited by ligand-induced and constitutive PPARγ activation [19]. The transcriptional mechanisms responsible for the suppression of telomerase activity by PPARγ ligands involve an inhibition of Ets-1-dependent transactivation of the TERT promoter [19]. Ets-1 is an early response gene that mediates a variety of growth signals in neointimal SMC proliferation [74]; and atherosclerosis [75] and PPARγ ligands have been reported to inhibit Ets-1 expression [76]. The relevance of telomerase as target for PPARγ was further demonstrated in SMCs overexpressing telomerase, in which the efficacy of PPARγ ligand pioglitazone to inhibit cell proliferation is lost [19]. These studies indicate that telomerase constitutes an important molecular target for the antiproliferative effects of PPARγ activation in SMCs.

### 3.4. Ligand-receptor relationship and specificity: is TZD-regulated gene expression in SMCs PPARγ-dependent?

Although the above-described evidence outlines the ability of TZD to suppress SMC proliferation and induce apoptosis, it remains controversial whether the cell-cycle-inhibitory effects of TZD occur through a ligand-dependent activation of PPARγ. Several experimental approaches have been used by different investigators to specifically address this question, including PPARγ-deficient cells [48, 77], overexpression of either dominant-negative or constitutively-active PPARγ mutants [19, 50, 53, 78], or pharmacologic inhibition of PPARγ [16, 53, 54, 61]. In PPARγ-deficient embryonic stem cells, TZD have been demonstrated to inhibit cell proliferation, which indicated that this effect might occur independent of their binding and activation of PPARγ [77]. In contrast to these earlier studies in stem cells, overexpression of a dominant-negative PPARγ mutant has been demonstrated to increase SMC proliferation in vitro and neointima formation in vivo (discussed in Section 4.1) pointing to a role of PPARγ to function as an endogenous repressor of SMC proliferation [78]. Complementary to these observations, overexpression of a constitutively-active PPARγ induces SMC apoptosis in the absence of ligand [53] while pharmacologic inhibition of PPARγ prevents rosiglitazone-induced apoptosis of neointimal SMCs [16]. In addition, many of the target genes, thought to be involved in the regulation of SMC proliferation/apoptosis by PPARγ ligands, have been demonstrated to be either directly regulated by overexpression of PPARγ or the ligand effect is reversed following pharmacologic inhibition of PPARγ [16, 19, 48, 50, 53, 54, 61, 78, 79]. These studies in concert support the concept that the antiproliferative activity of PPARγ ligands against SMC stems at least in part from a ligand-dependent activation of the receptor. However, further studies including in particular SMC-specific PPARγ-deficiency or overexpression are warranted to further support this notion.

A second important question that arises from this discussion relates to ligand specificity and whether the inhibition of SMC proliferation by agonists for PPARγ is exclusively mediated through this receptor or whether PPARγ ligands may also activate PPAR*α* or δ. Approximately 80% of the 34 residues defining the ligand binding cavity of PPARγ are conserved across the three receptor isotypes [11, 20]. In addition, all three isoforms possess unusually large binding pockets, compared to other nuclear receptors, which accommodate a diverse set of lipophilic acids as ligands [80]. Furthermore, anti-proliferative effects of PPARγ ligands are observed at concentrations considerably higher than their EC_50_ for transcriptional activation in cell-based transfection assays or in in vitro binding assays with isolated ligand-binding domain fragments [15, 81]. Considering this knowledge, at high concentrations spillover of PPARγ-selective ligands to PPAR*α* and/or PPARδ is theoretically possible and the antiproliferative activity of TZD observed in PPARγ-deficient cells could be explained by their binding to and activation of PPARα or PPARδ. Indeed, activation of PPARα represses SMC proliferation [82], while PPARδ activation has been reported to stimulate rather than inhibit growth of SMCs [83] and keratinocytes [84]. Although very few studies have directly compared the effects of PPARγ, PPARα, and PPARδ ligands on SMC function, Lin et al. recently identified that the above-described IRF-1-dependent apoptosis induced by PPARγ ligands is selective and not observed with PPARα or PPARδ ligands [61]. This study supports ligand selectivity for PPARγ in SMCs, although detailed studies are required to further address this question.

## 4. TZD IN THE TREATMENT OF CARDIOVASCULAR DISEASE

### 4.1. Lessons from animal models

TZD PPARγ ligands have been demonstrated to prevent the development of atherosclerosis in several murine atherosclerosis models including the low-density lipoprotein receptor-deficient (LDLR^−/−^) and the apolipoprotein E deficient mouse model (apoE^−/−^) [85–88]. This preventive effect on hyperlipidemia-induced atherosclerosis occurs independently of changes in circulating lipids, blood pressure, glucose, or insulin, implicating direct pleiotropic effects on the vascular wall. Inhibition of atherosclerosis by TZD ligands in these models appears to be also independent of their efficacy to improve insulin sensitivity as the prevention of atherosclerosis is observed in both insulin-sensitive and insulin-resistant ${\text{models}}^{\cdot }$ [85–88]. The mechanisms responsible for the prevention of atherosclerosis by TZD in these murine atherosclerosis models likely involve macrophage-driven processes contributing to atherosclerosis since conditional deletion of PPARγ in macrophages accelerates atherosclerosis [89]. In addition, specific deletion of PPARγ in EC has recently been demonstrated to increase blood pressure in mice suggesting that PPARγ in EC is an important regulator of hypertension, which may contribute to the prevention of atherosclerosis in murine models [90].

Consistent with the observations that TZD PPARγ ligands limit SMC proliferation in vitro, Law et al. demonstrated over a decade ago that the TZD ligand troglitazone reduces intimal hyperplasia in a rat carotid artery balloon injury model [91]. Subsequent studies confirmed these observations and demonstrated that TZD inhibit intimal hyperplasia in models of restenosis in both insulin-resistant and insulin-sensitive animals [92–95]. Similarly, Joner et al. recently demonstrated the prevention of in-stent restenosis by TZD ligands using a hypercholesterolemic rabbit atherosclerosis model [96]. Additional beneficial effects of TZD in the process of neointima formation include accelerated reendothelialization, which is mediated through an enhanced differentiation of angiogenic progenitor cells into mature endothelial cells [97, 98]. As detailed above, the question as to whether the prevention of neointima formation by TZD involves a receptor-dependent pathway has been addressed in a recent study using overexpression of PPARγ. While in vivo transfer of an adenoviral vector expressing wild-type PPARγ inhibited SMC proliferation and reduced neointima formation after balloon injury, overexpression of a dominant-negative PPARγ mutant increased neointima formation [78]. These studies have provided the first in vivo evidence to support a direct role of PPARγ in suppressing the proliferative response following vascular injury.

### 4.2. Clinical evidence for vascular protection by TZD

#### 4.2.1. Carotid artery intima/media thickness

Carotid artery intima/media thickness (CIMT) is a well-described surrogate marker for cardiovascular risk and correlates not only with the presence of cardiovascular risk factors but also with the risk of future macrovascular events [99, 100]. The first study that used CIMT to assess whether TZD treatment prevents the progression of atherosclerosis was performed 10 years ago. In this study 57 patients with type 2 diabetes were treated with 400 mg troglitazone, which resulted in a significant decline in CIMT after 3 months of treatment [101]. This reduction in CIMT with troglitazone has been confirmed in a recent cohort of patients with insulin-requiring type 2 diabetes [102]. A similar decline in CIMT was observed a few years later in two independent studies performed with pioglitazone [103, 104]. The recently reported CHICAGO trial (Carotid Intima-Media Thickness in Atherosclerosis Using Pioglitazone) was a randomized, double-blind, comparator-controlled, multicenter trial in patients with type 2 diabetes assessing the effect of pioglitazone versus the sulfonylurea glimepiride on CIMT progression [105]. In this study of 462 patients the primary endpoint of progression of mean CIMT was less with pioglitazone versus glimepiride after 72 weeks. Notably, the beneficial effect of pioglitazone on mean CIMT was similar across prespecified subgroups based on age, sex, systolic blood pressure, duration of type 2 diabetes, body mass index, HbA(1c) value, and statin use. The fourth CIMT study performed with pioglitazone compared the effects of pioglitazone (45 mg/d) and glimepiride (2.7 +/− 1.6 mg/d) in a randomized controlled study of 173 patients with type 2 diabetes [106]. In this study, CIMT was reduced only in the pioglitazone group and not in patients treated with glimepiride and this effect was independent of glycemic control.

Comparable results on CIMT progression have been obtained with rosiglitazone. Sidhu et al. analyzed the effect of rosiglitazone on CIMT progression in a double-blind, placebo-controlled randomized study in 92 non-diabetic patients with documented coronary artery disease [107]. In this study, rosiglitazone therapy revealed a reduced progression in CIMT after 48 weeks of treatment. The Rosiglitazone Atherosclerosis Study analyzed the effect of TZD treatment on CIMT progression in a mixed patient cohort of 555 subjects with type 2 diabetes or insulin resistance [108]. Although in this study there was no effect of rosiglitazone treatment in the mixed population of type 2 diabetes and insulin resistance, in the subanalysis of type 2 diabetic patients there was a reduced progression of CIMT. A third study reported by Stocker et al. analyzed whether rosiglitazone compared to metformin decreased CIMT in 93 subjects with type 2 diabetes [109]. In this study, metformin and rosiglitazone treatment led to similar improvement in glycemic control; however, CIMT progressed in the metformin group while regression of maximal CIMT was observed in the rosiglitazone group.

#### 4.2.2. Postangioplasty restenosis

Takagi et al. [110–112] first demonstrated that troglitazone reduced neointimal tissue proliferation after coronary stent implantation in patients with type 2 diabetes mellitus. Following the withdrawal of troglitazone from the market, it was subsequently demonstrated that pioglitazone has similar effects and significantly reduces neointimal tissue proliferation in patients with type 2 diabetes mellitus [113]. In this study, 44 patients with type 2 diabetes and 44 stented lesions were randomized to either pioglitazone therapy or control. Intravascular ultrasound demonstrated that the neointimal index in the pioglitazone group was significantly smaller than that in the control group. Similarly, Nishio et al. observed that the late luminal loss and in-stent restenosis were significantly less in patients treated with pioglitazone [114]. A third study performed with pioglitazone demonstrated in a randomized, placebo-controlled, double-blind trial that pioglitazone significantly reduced neointima volume after coronary stent implantation in patients without diabetes [115].

Comparable results have been obtained with rosiglitazone in a prospective, randomized, case-controlled trial involving 95 diabetic patients with coronary artery disease, which demonstrated that the in-stent restenosis rate was significantly reduced in the rosiglitazone group compared with the control group [116]. However, a second study of a smaller cohort of sixteen patients did not observe a significant decrease in in-stent luminal diameter stenosis measured by quantitative coronary angiography intravascular ultrasound [117]. Finally, the third study performed by Wang et al. suggested that the occurrence of coronary events following angioplasty in 71 patients was significantly decreased in the rosiglitazone group at 6-month follow-up [118]. These studies in concert suggest that TZD therapy in patients undergoing coronary stent implantation may be associated with less in-stent restenosis and repeated revascularization. This notion is further supported by two recent meta-analyses [119, 120]. However, decisions on clinical use of an adjunctive TZD therapy following coronary interventions must await larger double-blind clinical trials.

#### 4.2.3. Cardiovascular outcome studies

The beneficial vascular effects observed with TZD provided the rationale for larger cardiovascular trials and the first results from these studies are beginning to emerge. The Prospective Pioglitazone Clinical Trial in Macrovascular Events (PROactive) trail is a prospective, randomized controlled trial in 5238 patients with type 2 diabetes who had evidence of macrovascular disease [121]. This study tested theeffects of pioglitazone or placebo in addition to their glucose-lowering drugs and other medications on a combined vascular endpoint in patients with known vascular disease. The broad primary endpoint (the composite of all-cause mortality, nonfatal myocardial infarction (including silent myocardial infarction), stroke, acute coronary syndrome, endovascular or surgical intervention in the coronary or leg arteries, and amputation above the ankle) was not statistically different between the pioglitazone and placebo arm of the study. However, the study demonstrated a significant 16% reduction of the main cardiovascular secondary endpoint of all-cause mortality, myocardial infarction, and stroke in type 2 diabetic patients treated with pioglitazone. A recently published subanalysis out of this study further reported the effect of pioglitazone on recurrent myocardial infarction in 2,445 patients with type 2 diabetes and previous myocardial infarction [122]. In this prespecified endpoint, pioglitazone had a statistically significant beneficial effect on fatal and nonfatal MI (28% risk reduction) and acute coronary syndrome (37% risk reduction). A second subanalysis from the PROactive trial in patients with previous stroke (n=486 in the pioglitazone group and n=498 in the placebo group) further reported that pioglitazone reduced fatal or nonfatal stroke by 47% [123]. Consistent with the reported side-effect profile for TZD, the PROactive trial confirmed an increased rate of edema and heart failure in patients treated with pioglitazone [121]. However, in this context it is important to note that heart failure was a non-adjudicated event and mortality due to heart failure was not increased compared to the placebo group.

Currently, trials with rosiglitazone are being performed to determine whether rosiglitazone affects cardiovascular outcomes. Three clinical trials are currently testing approaches that use rosiglitazone to reduce cardiovascular disease in patients with diabetes: the Action to Control Cardiovascular Risk in Diabetes (ACCORD) trial [124], the Bypass Angioplasty Revascularization Investigation 2 Diabetes (BARI 2D) trial [125], and The Rosiglitazone Evaluated for Cardiac Outcomes and Regulation of Glycemia in Diabetes (RECORD) trial [126]. A recent meta-analysis of trials performed with rosiglitazone reported an association with a significant increase in the risk of myocardial infarction and with a nonsignificant increase of the risk of death from cardiovascular causes [127]. However, the authors of this meta-analysis acknowledged considerable limitations of their analysis and the National Institutes of Health (supporting the ACCORD and BARI 2D trials) found no evidence in this analysis to require discontinuing the use of rosiglitazone in the trials or to revise the study protocols [128]. Similarly, an interim analysis of the RECORD trial did not show a statistically significant difference between the rosiglitazone group and the control group for the endpoints acute myocardial infarction and death from cardiovascular causes, although patients treated with rosiglitazone were at increased risk to develop heart failure [129]. Therefore, completion of these studies will enable the determination whether rosiglitazone provides a similar reduction in cardiovascular outcomes as seen with pioglitazone and will aid to determine the most appropriate combination therapies for patients with type 2 diabetes.

## 5. SUMMARY AND CONCLUSIONS

Research performed over the last decade has highlighted an important role for TZD-induced PPARγ activation in vascular cells. TZD exert a broad spectrum of anti-inflammatory and anti-proliferative on all cell types participating in the development of cardiovascular diseases. A wealth of evidence from preclinical and clinical studies supports that these pleiotropic effects of TZD translate into reduced atherosclerosis and failure of coronary angioplasty as the primary approach to treat luminal obstruction. The PROactive trial was the first cardiovascular outcome trial to demonstrate that pioglitazone decreases all-cause mortality, myocardial infarction, and stroke in patients with type 2 diabetes. Further studies including the ACCORD, RECORD, and BARI 2D trials will determine whether similar effects are seen with rosiglitazone and outline ideal treatment strategies to reduce cardiovascular disease in patients with type 2 diabetes.
